# Trastuzumab Provides a Comparable Prognosis in Patients With HER2-Positive Breast Cancer to Those With HER2-Negative Breast Cancer: *Post Hoc* Analyses of a Randomized Controlled Trial of Post-Mastectomy Hypofractionated Radiotherapy

**DOI:** 10.3389/fonc.2020.605750

**Published:** 2021-01-26

**Authors:** Guang-Yi Sun, Hao Jing, Shu-Lian Wang, Yong-Wen Song, Jing Jin, Hui Fang, Yue-Ping Liu, Hua Ren, Yu Tang, Xu-Ran Zhao, Yu-Chun Song, Si-Ye Chen, Zhuan-Bo Yang, Bo Chen, Yuan Tang, Ning Li, Ning-Ning Lu, Shu-Nan Qi, Yong Yang, Ye-Xiong Li

**Affiliations:** Department of Radiation Oncology, National Cancer Center/National Clinical Research Center for Cancer/Cancer Hospital, Chinese Academy of Medical Sciences and Peking Union Medical College, Beijing, China

**Keywords:** breast cancer, HER2, trastuzumab, radiotherapy, prognosis

## Abstract

**Background and Purpose:**

We investigated the locoregional effect of trastuzumab, and determined whether patients with human epidermal growth factor receptor (HER)2-positive breast cancer (BC) treated with trastuzumab could achieve comparable efficacy to that of patients with HER2-negative BC.

**Materials and Methods:**

This was *post hoc* analyses of data of 793 BC patients from a randomized controlled trial comparing post-mastectomy hypofractionated radiotherapy with conventional fractionated radiotherapy. Survival rates were analyzed by the Kaplan–Meier method and compared by the log-rank test.

**Results:**

Patients were classified into three groups: HER2-negative (HER2^−^; n = 547), HER2-positve with trastuzumab (HER2^+^ + T; n = 136), and HER2-positive without trastuzumab (HER2^+^ − T; n = 110). The HER2^+^ + T group had significantly lower locoregional recurrence (LRR, 6.0% *vs.* 13.9%), distant metastasis (DM, 17.4% *vs.* 33.8%) and higher disease-free survival (DFS, 81.2% *vs.* 61.9%) at 5 years than that of the HER2^+^ − T group (*P* <.05). The HER2^−^ group had significantly lower LRR (6.8% *vs.* 13.9%), DM (22.4% *vs.* 33.8%) and higher DFS (76.1% *vs.* 61.9%) at 5 years than that of the HER2^+^ − T group (*P* <.05). The difference in LRR, DM and DFS at 5 years was not significant between the HER2^+^ + T group and HER2^−^ group (*P* >.05). Different annual LRR patterns was found among groups according to HR status.

**Conclusion:**

Trastuzumab reduces LRR in patients with locally advanced HER2-positive BC who have received post-mastectomy radiotherapy. It provides comparable DFS to that with patients with HER2-negative BC.

## Introduction

Increased expression of human epidermal growth factor receptor (HER)2, one of the most important molecular markers for breast cancer (BC), occurs in 15–25% of women with BC, and is associated with a poor prognosis (1, 2). Several randomized trials have demonstrated adjuvant use of trastuzumab (Herceptin^®^; Roche, Basel, Switzerland) to have a major and persistent benefit in reducing the risk of distant recurrence and death in patients with HER2-positive BC (3–5). However, the locoregional benefit of trastuzumab has not been elucidated clearly, especially in patients who have received radiotherapy (6–8). Little is known about the locoregional effect of trastuzumab in patients with locally advanced BC treated with adjuvant radiotherapy. In addition, there are limited data on outcome comparisons between patients with HER2-positive disease in the setting of trastuzumab and patients with HER2-negative disease (9, 10).

We aimed to determine whether: (i) adjuvant trastuzumab reduced locoregional recurrence (LRR) in patients with stage-II and III BC (according to AJCC 7^th^ edition) treated with post-mastectomy radiotherapy; (ii) patients with HER2-positive BC treated with trastuzumab had comparable outcomes to those of patients with HER2-negative BC.

## Materials and Methods

### Patients

**Figure 1:**
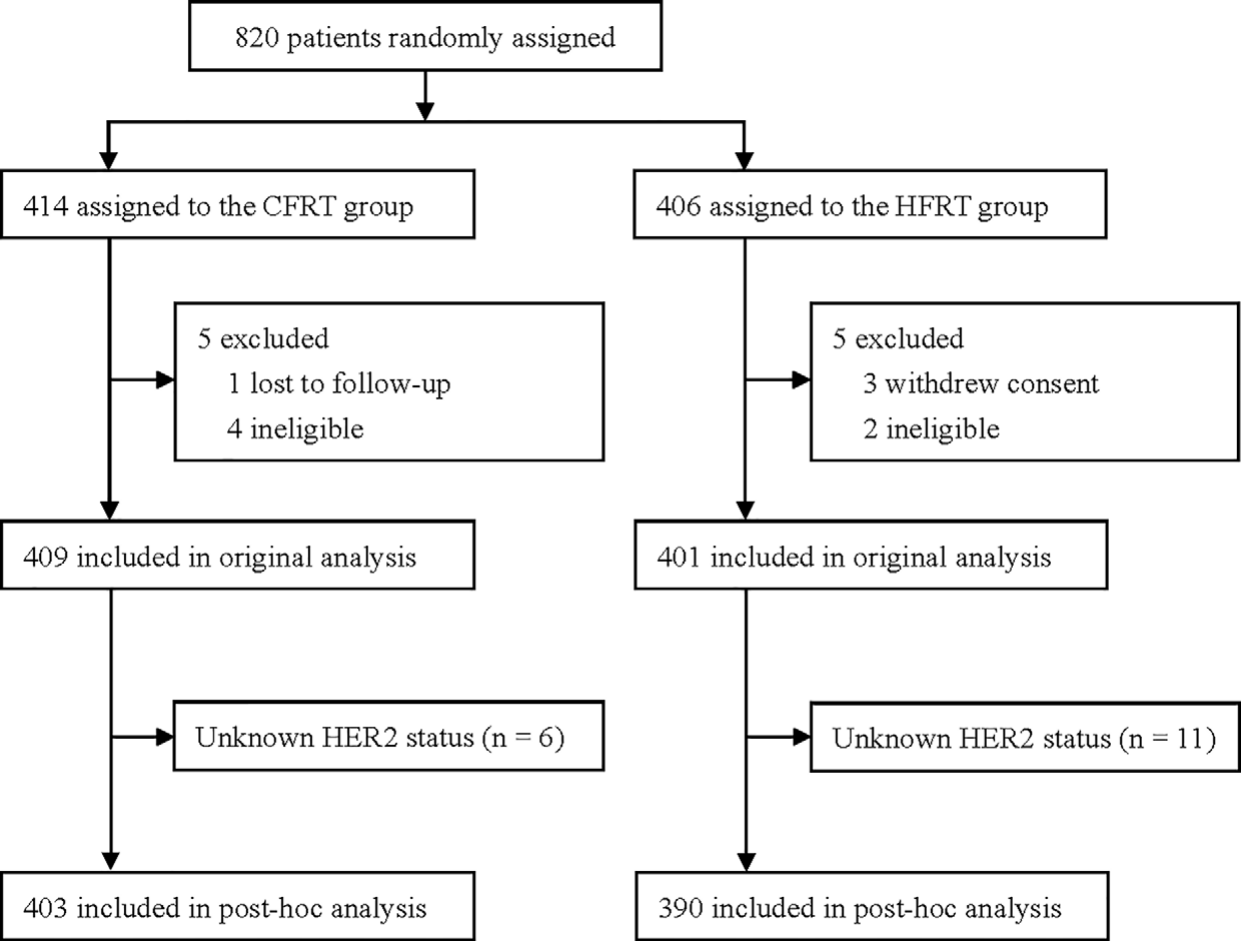
Trial profile. CFRT, conventional fractionated radiotherapy; HFRT, hypofractionated radiotherapy; HER2, human epidermal growth factor receptor 2.

This study was a *post hoc* analysis of data from a randomized clinical trial (NCT00793962) conducted between 2008 and 2016. The original trial recruited 820 women aged 18–75 years who had undergone mastectomy for high-risk BC with negative margins (i.e., ≥4 positive axillary lymph nodes or a primary tumor of stage T3–4) and compared post-mastectomy hypofractionated radiotherapy (HFRT, 43.5 Gy in 15 fractions over 3 weeks) with conventional fractionated radiotherapy (CFRT, 50 Gy in 25 fractions over 5 weeks). The cohort has been described in detail elsewhere^11^. Patients were designated as “HER2-positive” if they demonstrated a membranous staining score of 3+ on immunohistochemical (IHC) analyses, or gene amplification on fluorescence *in situ* hybridization (FISH). HER2 copy number that was not amplified by FISH or read as 0–1+ by IHC was designated as “HER2-negative”. Patients who were IHC 2+ without a confirmatory FISH result were excluded from analyses (n = 17). Other clinical and histologic information of these patients was collected from case records. The final follow-up was November 2019. A total of 793 patients were included for evaluation (**Figure 1**).

### Statistical Analyses

The relationship between HER2 status and outcomes was analyzed. “LRR” was defined as the first recurrence after surgery in the ipsilateral chest wall or axillary, supra-/infraclavicular, or internal mammary nodal regions regardless of the status of systemic disease. “Distant metastasis” (DM) was defined as the first recurrence after surgery in any distant site. Overall survival (OS) was calculated from the date of the surgical procedure to the date of death due to any cause or final follow-up. Disease-free survival (DFS) was calculated from the date of surgery to date of recurrence or death, or final follow-up.

Statistical analyses were undertaken using SPSS v23.0 (IBM, Armonk, NY, USA) and R v3.5.3 (R Project for Statistical Computing, Austria, Vienna; www.r-project.org/). Categorical variables were summarized as frequencies and percentages, and were compared using the Fisher exact test or chi-square test.

The relapse rate and survival rate were calculated using the Kaplan–Meier method and compared using the log-rank test (including pairwise comparisons). The hazard rate of LRR was computed using the “muhaz” package in R. The association of potential prognostic factors with survival outcomes was tested by univariate Cox regression analysis. Factors associated with outcome in the univariate analysis (at P <.05) were entered into multivariable proportional hazards regression analysis to identify independent predictors. Statistical significance was set at P ≤.05. Bonferroni’s correction for significance of association was applied to compensate for multiple comparisons.

## Results

### Patient Characteristics

**Table 1** shows the demographic, tumor, and treatment characteristics of the 793 patients for whom HER2 status was identified. The median age of the study cohort was 49 (range, 24–74) years. All patients underwent mastectomy and radiotherapy. All patients received a median of six (range, 4–11) cycles of chemotherapy (neoadjuvant and/or adjuvant).

**Table 1:** Baseline characteristics of the 793 patients with breast cancer.

	Total; n (%)	HER2-negative; n (%)	HER2-positive		*P*
			With trastuzumab; n (%)	Without trastuzumab; n (%)	
Treatment period (years)					.005
2008–2013	566 (71.4)	388 (70.9)	87 (64.0)	91 (82.7)	
2014–2016	227 (28.6)	159 (29.1)	49 (36.0)	19 (17.3)	
Age (years)					.457
<40	139 (17.5)	95 (17.4)	28 (20.6)	16 (14.5)	
≥40	654 (82.5)	452 (82.6)	108 (79.4)	94 (85.5)	
Stage					.669
II–IIIA	498 (62.8)	346 (63.3)	81 (59.6)	71 (64.5)	
IIIB–IIIC	295 (37.2)	201 (36.7)	55 (40.4)	39 (35.5)	
Lymphovascular invasion					.842
No	531 (67.0)	363 (66.4)	92 (67.6)	76 (69.1)	
Yes	262 (33.0)	184 (33.6)	44 (32.4)	34 (30.9)	
Histology grade					.166
I–II	501 (63.2)	353 (64.5)	86 (63.2)	62 (56.4)	
III	227 (28.6)	145 (26.5)	44 (32.4)	38 (34.5)	
Unknown	65 (8.2)	49 (9.0)	6 (4.4)	10 (9.1)	
Hormonal receptor					<.001
Negative	151 (19.0)	75 (13.7)	47 (34.6)	29 (26.4)	
Positive	642 (81.0)	472 (86.3)	89 (65.4)	81 (73.6)	
Neoadjuvant chemotherapy					.224
No	591 (74.5)	417 (76.2)	98 (72.1)	76 (69.1)	
Yes	202 (25.5)	130 (23.8)	38 (27.9)	34 (30.9)	
Radiation fractionation					.093
HFRT	390 (49.2)	255 (46.6)	76 (55.9)	59 (53.6)	
CFRT	403 (50.8)	292 (53.4)	60 (44.1)	51 (46.4)	
Hormonal therapy					<.001
No	187 (23.6)	101 (18.5)	54 (39.7)	32 (29.1)	
Yes	559 (70.5)	412 (75.3)	79 (58.1)	68 (61.8)	
Unknown	47 (5.9)	34 (6.2)	3 (2.2)	10 (9.1)	

HER2, human epidermal growth factor receptor 2; CFRT, conventional fractionated radiotherapy; HFRT, hypofractionated radiotherapy.

According to HER2 status and trastuzumab treatment, patients were classified into three groups: HER2-negative (HER2^−^; n = 547, 69.0%), HER2-positive with trastuzumab treatment (HER2^+^ + T; n = 136, 17.1%), and HER2-positive without trastuzumab treatment (HER2^+^ − T; n = 110, 13.9%).

The proportion of HER2-positive patients treated with trastuzumab increased from 31.6% in 2008 to 77.8% in 2016 (**Supplementary Figure 1**). As shown in **Table 1**, the HER2^+^ − T group had more patients treated between 2008 and 2013 compared with that in the HER2^−^ group and HER2^+^ + T group (Bonferroni-corrected *P* = .033 and Bonferroni-corrected *P* = .006, respectively). The proportion of patients with hormonal receptor (HR)-positive disease was significantly higher in the HER2^−^ group than that in the HER2^+^ + T group and HER2^+^ − T group (Bonferroni-corrected *P* <.001 and Bonferroni-corrected *P* = .003, respectively). As a result, significantly more patients in the HER2^−^ group received hormonal therapy than patients in the HER2^+^ + T group or HER2^+^ − T group (Bonferroni-corrected *P* <.001 and Bonferroni-corrected *P* = .018, respectively). All other characteristics were well balanced between the three groups.

### Outcomes of the Entire Cohort

**Figure 2:**
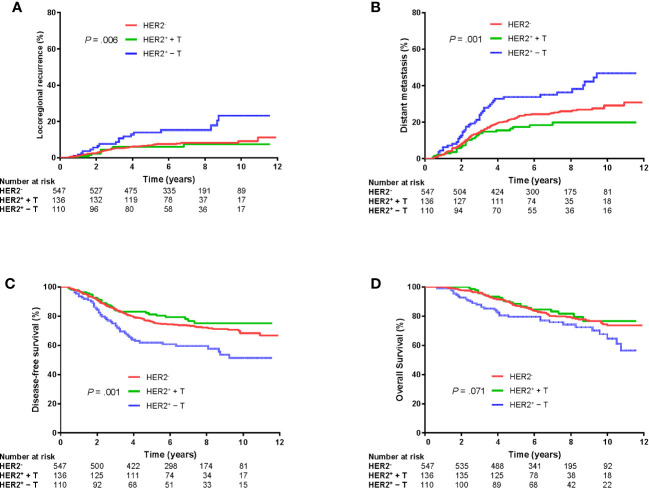
Kaplan–Meier plots of locoregional recurrence **(A)**, distant metastasis **(B)**, disease-free survival **(C)**, and overall survival **(D)** of patients grouped according to HER2 status and trastuzumab treatment. HER2^−^, HER2-negative; HER2^+^ + T, HER2-positive with trastuzumab; HER2^+^ − T, HER2-positive without trastuzumab.

Median follow-up was 6.8 (interquartile range: 5.0–9.1) years. LRR, DM, DFS, and OS rates at 5 years for the whole group was 7.6%, 23.1%, 75.1%, and 86.9%, respectively. There were significant differences in LRR, DM and DFS among HER2-, HER2^+^ + T, and HER2^+^ − T groups (*P* = .006, *P* = .001, *P* = .001, respectively; **Figures 2A–C**). However, there was no significant difference in OS among the three groups (*P* = .071, **Figure 2D**).

Further log-rank pairwise comparisons showed the HER2^+^ + T group had significantly lower LRR, DM and higher DFS at 5 years than that in the HER2^+^ − T group (6.0% *vs.* 13.9%, Bonferroni-corrected *P* = .048; 17.4% *vs.* 33.8%, Bonferroni-corrected *P* = .003; 81.2% *vs.* 61.9%, Bonferroni-corrected *P* = .003; respectively). The HER2^−^ group also had significantly lower LRR, DM and higher DFS at 5 years than that in the HER2^+^ − T group (6.8% *vs.* 13.9%, Bonferroni-corrected *P* = .009; 22.4% *vs.* 33.8%, Bonferroni-corrected *P* = .009; 76.1% *vs.* 61.9%, Bonferroni-corrected *P* = .003; respectively). However, there were no significant differences in LRR (Bonferroni-corrected *P* = .663), DM (Bonferroni-corrected *P* = .357) or DFS (Bonferroni-corrected *P* = .810) at 5 years between the HER2^+^ + T group and HER2^−^ group.

Univariate and multivariate analyses of prognostic factors for LRR, DM, DFS and OS are shown in **Tables 2** and **3**. In multivariate analyses, the combination of HER2 status and trastuzumab treatment was an independent prognostic factor for LRR (*P* = .011), DM (*P* = .002) and DFS (*P* <.001). Other independent prognostic factors were the treatment period, age, stage, neoadjuvant chemotherapy, and hormonal therapy.

**Table 2:** Univariate analysis of prognostic factors for locoregional recurrence, distant metastasis, disease-free survival and overall survival.

Characteristic	LRR		DM		DFS		OS	
	Hazard ratio(95%CI)	*P*	Hazard ratio(95%CI)	*P*	Hazard ratio(95%CI)	*P*	Hazard ratio(95%CI)	*P*
Treatment period (years)								
2008–2013	1.00		1.00		1.00		1.00	
2014–2016	0.55 (0.29–1.05)	.066	0.63 (0.44–0.90)	.010	0.62 (0.44–0.88)	.006	0.62 (0.39–0.98)	.041
Age (years)								
<40	1.00		1.00		1.00		1.00	
≥40	0.70 (0.40–1.22)	.213	0.60 (0.44–0.82)	.002	0.62 (0.46–0.85)	.002	0.77 (0.53–1.12)	.167
Stage								
II–IIIA	1.00		1.00		1.00		1.00	
IIIB–IIIC	1.58 (0.99–2.53)	.055	1.99 (1.52–2.62)	<.001	1.98 (1.53–2.57)	<.001	2.26 (1.66–3.08)	<.001
Lymphovascular invasion								
No	1.00		1.00		1.00		1.00	
Yes	1.54 (0.96–2.48)	.077	1.26 (0.95–1.67)	.110	1.24 (0.95–1.63)	.112	1.38 (1.01–1.90)	.045
Histology grade								
I–II	1.00		1.00		1.00		1.00	
III	1.17 (0.69–1.98)	.562	1.01 (0.74–1.38)	.932	1.05 (0.78–1.41)	.738	1.33 (0.94–1.87)	.101
Hormonal receptor								
Negative	1.00		1.00		1.00		1.00	
Positive	0.57 (0.33–0.96)	.034	0.75 (0.54–1.04)	.080	0.72 (0.53–0.98)	.039	0.51 (0.36–0.72)	<.001
Neoadjuvant chemotherapy								
No	1.00		1.00		1.00		1.00	
Yes	1.65 (1.01–2.71)	.047	1.41 (1.05–1.89)	.024	1.43 (1.08–1.90)	.014	1.59 (1.15–2.21)	.005
Radiation fractionation								
HFRT			1.00		1.00		1.00	
CFRT	0.80 (0.50–1.29)	.363	1.09 (0.83–1.43)	.531	1.07 (0.83–1.39)	.60	1.06 (0.78–1.44)	.713
Hormonal therapy								
No	1.00		1.00		1.00		1.00	
Yes	0.46 (0.28–0.77)	.003	0.79 (0.51–0.94)	.019	0.66 (0.49–0.88)	.005	1.06 (0.99–1.14)	<.001
HER2 and trastuzumab		.008		.001		.001		.089
HER2^−^	1.00		1.00		1.00		1.00	
HER2^+^ + T	0.86 (0.42–1.77)	.681	0.72 (0.47–1.10)	.125	0.81 (0.54–1.19)	.277	0.90 (0.57–1.41)	.638
HER2^+^ − T	2.28 (1.31–3.95)	.003	1.67 (1.18–2.35)	.003	1.73 (1.25–2.40)	.001	1.52 (1.03–2.26)	.037

LRR, locoregional recurrence; DM, distant metastasis; DFS, disease-free survival; OS, overall survival; HFRT, hypofractionated radiation therapy; CFRT, conventional fractionated radiation therapy; HER2, human epidermal growth factor receptor 2; HER2^−^, HER2-negative; HER2^+^ + T, HER2-positive with trastuzumab; HER2^+^ − T, HER2-positive without trastuzumab.

**Table 3:** Multivariate analysis of prognostic factors for locoregional recurrence, distant metastasis, disease-free survival and overall survival.

Characteristics	LRR		DM		DFS		OS	
	Hazard ratio(95%CI)	*P*	Hazard ratio(95%CI)	*P*	Hazard ratio(95%CI)	*P*	Hazard ratio(95%CI)	*P*
Treatment period (years)								
2008–2013	1.00		1.00		1.00		1.00	
2014–2016	0.56 (0.28–1.11)	.095	0.67 (0.46–0.98)	.039	0.67 (0.47–0.97)	.033	0.67 (0.50–1.11)	.106
Age (years)								
<40	1.00		1.00		1.00		1.00	
≥40	0.63 (0.35–1.11)	.109	0.57 (0.41–0.79)	.001	0.58 (0.42–0.80)	.001	0.75 (0.50–1.11)	.151
Stage								
II–IIIA	1.00		1.00		1.00		1.00	
IIIB–IIIC	1.41 (0.85–2.33)	.180	1.97 (1.47–2.64)	<.001	2.00 (1.51–2.65)	<.001	2.27 (1.62–3.17)	<.001
Lymphovascular invasion								
No	1.00		1.00		1.00		1.00	
Yes	1.48 (0.87–2.50)	.147	1.17 (0.86–1.60)	.312	1.19 (0.88–1.61)	.245	1.29 (0.90–1.84)	.159
Hormonal receptor								
Negative	1.00		1.00		1.00		1.00	
Positive	1.54 (0.62–3.78)	.351	0.62 (0.35–1.13)	.796	1.06 (0.58–1.93)	.849	0.95 (0.49–1.85)	.876
Neoadjuvant chemotherapy								
No	1.00		1.00		1.00		1.00	
Yes	1.41 (0.82–2.42)	.213	1.33 (0.97–1.84)	.078	1.39 (1.02–1.89)	.036	1.70 (1.19–2.42)	.003
Hormonal therapy								
No	1.00		1.00		1.00		1.00	
Yes	0.33 (0.14–0.75)	.009	0.62 (0.35–1.13)	.118	0.61 (0.35–1.07)	.087	0.44 (0.23–0.84)	.013
HER2 and trastuzumab		.011		.002		<.001		.338
HER2^−^	1.00		1.00		1.00		1.00	
HER2^+^ + T	0.76 (0.36–1.58)	.458	0.65 (0.42–1.00)	.054	0.68 (0.45–1.03)	.068	0.67 (0.41–1.08)	.105
HER2^+^ − T	2.18 (1.22–3.90)	.008	1.61 (1.12–2.32)	.010	1.71 (1.21–2.43)	.003	1.38 (0.91–2.10)	.134

LRR, locoregional recurrence; DM, distant metastasis; DFS, disease-free survival; OS, overall survival; HER2, human epidermal growth factor receptor 2; HER2^−^, HER2-negative; HER2^+^ + T, HER2-positive with trastuzumab; HER2^+^ − T, HER2-positive without trastuzumab.

### Outcomes According to HR Status

**Figure 3:**
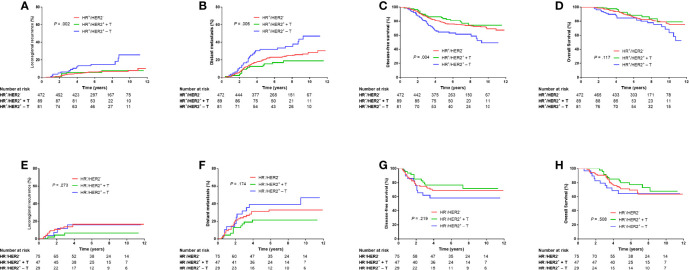
Kaplan–Meier plots of locoregional recurrence **(A, E)**, distant metastasis **(B, F)**, disease-free survival **(C, G)**, and overall survival **(D, H)** of HR-positive patients and HR-negative patients grouped according to HER2 status and trastuzumab treatment. HR^−^, hormonal receptor-negative; HR^+^, hormonal receptor-positive; HER2^−^, HER2-negative; HER2^+^ + T, HER2-positive with trastuzumab; HER2^+^ − T, HER2-positive without trastuzumab.

When HR was positive, the HER2^−^ group and HER2^+^ + T group had significantly lower LRR (5.3% *vs.* 13.2%, Bonferroni-corrected *P* = .003; 5.7% *vs.* 13.2%, Bonferroni-corrected *P* = .044; **Figure 3A**), DM (21.0% *vs.* 31.8%, Bonferroni-corrected *P* = .027; 15.3% *vs.* 31.8%, Bonferroni-corrected *P* = .012; **Figure 3B**) and higher DFS (77.7% *vs.* 64.7%, Bonferroni-corrected *P* = .009; 83.7% *vs.* 64.7%, Bonferroni-corrected *P* = .012; **Figure 3C**) at 5 years than that in the HER2^+^ − T group. There were no significant differences in LRR (Bonferroni-corrected *P* = .897, **Figure 3A**), DM (Bonferroni-corrected *P* = .489, **Figure 3B**) or DFS (Bonferroni-corrected *P* = .870, **Figure 3C**) at 5 years between the HER2^−^ group and HER2^+^ + T group.

When HR was negative, there were no significant differences in LRR, DM, DFS or OS among HER^−^, HER2^+^ + T, or HER2^+^ − T groups (*P* = .273, *P* = .174, *P* = .219, *P* = .508, respectively; **Figures 3E–H**).

### Annual Hazard Rate of LRR

**Figure 4:**
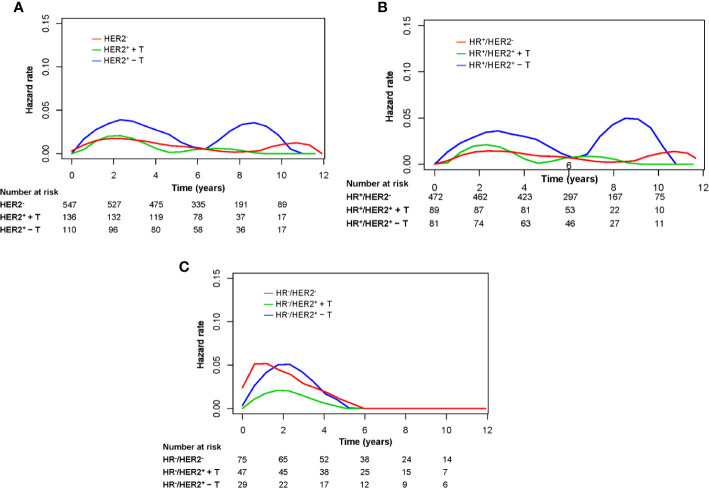
Annual hazard rates for locoregional recurrence of the entire group **(A)**, HR-positive patients **(B)**, and HR-negative patients **(C)** grouped according to HER2 status and trastuzumab treatment. HR^−^, hormonal receptor-negative; HR^+^, hormonal receptor-positive; HER2^−^, HER2-negative; HER2^+^ + T, HER2-positive with trastuzumab; HER2^+^ − T, HER2-positive without trastuzumab.

**Figure 4** shows the annual LRR patterns of the three groups in the entire cohort, HR^+^ cohort, and HR^–^ cohort. Visual inspection of the LRR hazard curves showed a difference in LRR patterns between the HER2^–^ group and HER2^+^ + T group and HER2^+^ – T group. In the entire cohort, the annual hazard rate of LRR in the HER2^+^ – T group was consistently higher than that in the HER2^–^ group or HER2^+^ + T group. The annual LRR curve of the HER2^+^ – T group displayed an obvious double-peaked pattern, with an early peak at ~2.5 years and a late peak at ~9 years. The annual LRR curve of the HER2^+^ + T group displayed a single early peak at ~2.5 years. The annual LRR curve of the HER2^−^ group displayed a continuously low risk without an obvious peak (**Figure 4A**). The observed difference in LRR among these three groups occurred mainly during the first 5 years rather than beyond 5 years. The annual LRR rate of HER2^–^, HER2^+^ + T, and HER2^+^ – T groups was 1.4%, 1.3% and 3.0% during the first 5 years (*P* = .049), and 0.5%, 0.4% and 1.9% after 5 years (*P* = .070), respectively.

The annual LRR pattern of the three groups in the HR^+^ cohort was similar to that of the entire cohort (**Figure 4B**). In the HR^+^ cohort, the annual LRR rate of HER2^−^, HER2^+^ + T, and HER2^+^ − T groups was 1.1%, 1.2% and 2.8% during the first 5 years (*P* = .042), and 0.6%, 0.6% and 2.4% after 5 years (*P* = .082), respectively. In the HR^−^ cohort, all three groups showed a single early LRR peak, at ~1 year for the HER2^−^ group, and at ~2 years for HER2^+^ + T and HER2^+^ − T groups (**Figure 4C**). The annual LRR rate of HER2^−^, HER2^+^ + T, and HER2^+^ − T groups was 3.9%, 1.4% and 3.7% during the first 5 years (*P* = .240), and 0.0%, 0.0% and 0.0% after 5 years, respectively.

## Discussion

Previously, we demonstrated that post-mastectomy HFRT was non-inferior to CFRT in high-risk BC patients (11). The present *post hoc* analyses showed that: (i) trastuzumab decreased the risk of LRR and improved DFS significantly in patients with HER2-positive disease who received post-mastectomy radiotherapy (PMRT); (ii) patients with HER2-positive disease treated with trastuzumab had a comparable prognosis to that of patients with HER2-negative disease.

Randomized trials have revealed survival and local-control benefits from PMRT in patients with high-risk BC (12–14). One meta-analysis concluded that PMRT reduced LRR and BC mortality, especially for patients with lymph-node involvement (15). Therefore, patients with stage-III BC who have undergone modified mastectomy have received PMRT routinely. However, those studies were conducted in the non-trastuzumab era, and whether there is a synergistic effect between trastuzumab and PMRT is not known. As trastuzumab treatment was not covered by the national health insurance plan during the period of this study, only half of the patients with HER2-positive breast cancer received anti-HER2 therapy. It gave us an opportunity to discuss these issues. In the present study, 93.8% of patients had stage-III BC and all received PMRT. We showed that the absolute reduction in LRR from trastuzumab treatment in HER2-positive patients was 8%.

The question regarding whether trastuzumab reduces LRR risk in patients with HER2-positive disease is controversial. Perterson et al. reported similar high locoregional recurrence-free survival of 98.3% among 404 patients who received trastuzumab and 97.8% in 344 patients with T1-2N0 BC who did not receive trastuzumab (16). However, a study by Keiss and colleagues also involving patients with T1-2N0 BC showed that locoregional recurrence-free survival after breast-conserving treatment was higher among the trastuzumab-treated group (99% *vs.* 90%, *P* = .01) (7). Furthermore, other studies have shown that trastuzumab reduced LRR risk among HER2-positive patients with HR-positive stage-I–III BC, whereas patients with HR-negative BC had persistently increased LRR risk despite trastuzumab use (6, 17, 18). The present study showed similar findings whereby trastuzumab significantly reduced LRR risk in a HR-positive cohort rather than a HR-negative cohort, however, the sample size in the HR-negative cohort was small. The small sample size might also explain the findings that the relapse and survival outcomes were similar between triple-negative and HR^-^HER2^+^ + T groups. In HER2-positive patients with stage-I–III BC who received mastectomy, Lanning et al. found that trastuzumab did not decrease LRR risk in patients who did not receive PMRT, but it decreased LRR risk among those who received PMRT, suggesting that the greatest benefit is seen in a higher-risk subset of patients (8). That finding was confirmed by Wang et al., who found that trastuzumab reduced the 5-year LRR rate (4.9% *vs.* 17.9%, *P* <.05) in HER2-positive patients treated with mastectomy and PMRT (19). Taken together, data suggest that trastuzumab may play an important part in reducing LRR risk among patients with more advanced disease and those with HR-positive disease.

Analyses of hazard function defines, in much greater detail, changes in LRR risk over time and highlights when recurrence occurs rather than simply calculating the overall recurrence risk. We observed a double-peaked pattern of LRR in HER2-positive patients who did not receive trastuzumab in the HR-positive cohort, and the second peak disappeared upon trastuzumab treatment. The treatment effect of trastuzumab on LRR was not proportional over time, and occurred mainly during the first 5 years rather than beyond 5 years. Previously, it has been reported that the hazard rate for tumor relapse of estrogen receptor (ER)-positive and ER-negative tumors displays a bimodal curve with a first, dominant early peak and a second, lower later surge followed by a long-lasting “tail” (20). A double-peaked pattern of relapse or death has been observed in BC patients regardless of ER status or HER2 status in the absence of trastuzumab (21). Ribelles and colleagues also observed this double-peaked pattern of relapse or death in a Luminal B and HER2-positive group (88% treated without trastuzumab), whereas a single early peak was noted in a triple-negative group (22). However, reports on LRR patterns are limited. The present study is the first to show different annual LRR patterns among groups stratified by trastuzumab treatment and according to HR status. In contrast to our findings that trastuzumab did not significantly reduce the risk of LRR beyond 5 years, a combined analysis for relapse or death by Chumsri and coworkers showed that the benefit of adjuvant trastuzumab therapy was seen in years 0 to 5 (hazard ratio, 0.42; P = .001) and years 5 to 10 (hazard ratio, 0.69; P = .03) (23). This difference might be explained by the different endpoints used in our study, or inadequate samples that prevented achievement of a significant difference in comparison beyond 5 years. Although little clinical data exist on the interaction of HR status and HER2 status on locoregional outcomes in the trastuzumab era, these findings indicated different surveillance strategies for different groups.

Randomized studies have established that trastuzumab improves the survival rates of patients with HER2-positive tumors (3–5, 23), whereas studies are limited for comparing the outcome of HER2-positive patients taking trastuzumab with that of HER2-negative patients. We found that HER2-positive patients taking trastuzumab had comparable DFS with that of HER2-negative patients. This finding has been corroborated by other studies. Fokter et al. reported no significant difference in DFS between trastuzumab-treated HER2-positive patients and HER2-negative patients (9). Qin et al found that the DFS difference at 5 years between HER2-negative patients and HER2-positive patients receiving trastuzumab was not significant (71.7% *vs.* 77.8%, *P* = .503) (10). Some studies on locally advanced BC or metastatic BC have shown that trastuzumab-treated HER2-positive patients have better event-free survival or OS than that in patients with HER2-negative BC (24, 25). The negative prognostic effect of HER2-positivity observed before trastuzumab treatment became available might disappear completely in the era of routine administration of trastuzumab. Therefore, the prognostic importance of HER2 status must be considered in the context of anti-HER2 treatment. In the recent eighth edition of the American Joint Committee on Cancer (AJCC) staging system for BC, the prognostic-stage groups showed that patients with HER2-positive tumors tended to be grouped into a lower stage than patients with HER2-negative tumors provided that other staging factors were identical because most of the patients used to generate the eighth edition of the AJCC staging system in the initial models received trastuzumab (26). For example, for a patient with T3N2M0, grade 3, ER-positive, and PR-positive BC, she was grouped into IIA if HER2 was positive, and IIB if HER2 was negative.

Our study had three main limitations. First, thanks to developments in diagnostic and therapeutic strategies, treatment guidelines for patients with BC have changed, and our study population may not reflect the outcomes for patients being treated currently. This study initiated originally from 2008, so we found the treatment period to be an independent predictor for DM and DFS. Nevertheless, the combination of HER2 status and trastuzumab treatment was demonstrated to be an independent predictor for LRR, DM, and DFS after adjustment for the treatment period. Second, because of the limited follow-up time, the findings on hazard rates over time warrant further validation. Third, this study was an unplanned *post hoc* analysis, patients taking and not taking trastuzumab were not assigned randomly, so a selection bias was unavoidable. We attempted to eliminate the influence of confounding factors in the multivariate analysis by including all other known prognostic factors. Nevertheless, this study is the first to assess the locoregional effect of trastuzumab in patients with locally advanced BC treated with adjuvant radiotherapy, and the findings on the LRR patterns over time merit further investigation.

## Conclusions

Trastuzumab reduces the risk of LRR in patients with locally advanced HER2-positive BC who have received PMRT. It provides comparable DFS in patients with HER2-positive disease to those with HER2-negative disease.

## Data Availability Statement

All datasets presented in this study are included in the article/**Supplementary Material**.

## Ethics Statement

The studies involving human participants were reviewed and approved by the Institutional Review Board of Cancer Hospital, Chinese Academy of Medical Sciences and Peking Union Medical College. The patients/participants provided their written informed consent to participate in this study.

## Author Contributions

G-YS and HJ: Formal analysis, investigation, data collection, methodology, and writing of first draft. Y-WS, JJ, HF, Y-PL, HR, YuT, BC, YuaT, NL, N-NL, S-NQ, YY: Patient care and review and editing of the manuscript. X-RZ, Y-CS, S-YC, Z-BY: Data collection and review of the original draft and editing. S-LW, Y-XL: Formal analysis and data collection, validation, statistics guidance, and project administration, patient care, and writing and editing of the first draft of the manuscript. All authors contributed to the article and approved the submitted version.

## Funding

This work was supported by grants from Beijing Marathon of Hope, Cancer Foundation of China (LC2012A11) and National Natural Science Foundation of China (81972860).

## Conflict of Interest

The authors declare that the research was conducted in the absence of any commercial or financial relationships that could be construed as a potential conflict of interest.

## Supplementary Material

Supplementary Figure 1 The trends of the proportion of HER2-positive patients treated with trastuzumab over the study period.
