# Supplementary figures and images for: Trastuzumab Provides a Comparable Prognosis in Patients With HER2-Positive Breast Cancer to Those With HER2-Negative Breast Cancer: Post Hoc Analyses of a Randomized Controlled Trial of Post-Mastectomy Hypofractionated Radiotherapy
Source: Front Oncol. 2021 Jan 26;10:605750. doi: 10.3389/fonc.2020.605750 (PMC7871908; doi:10.3389/fonc.2020.605750)

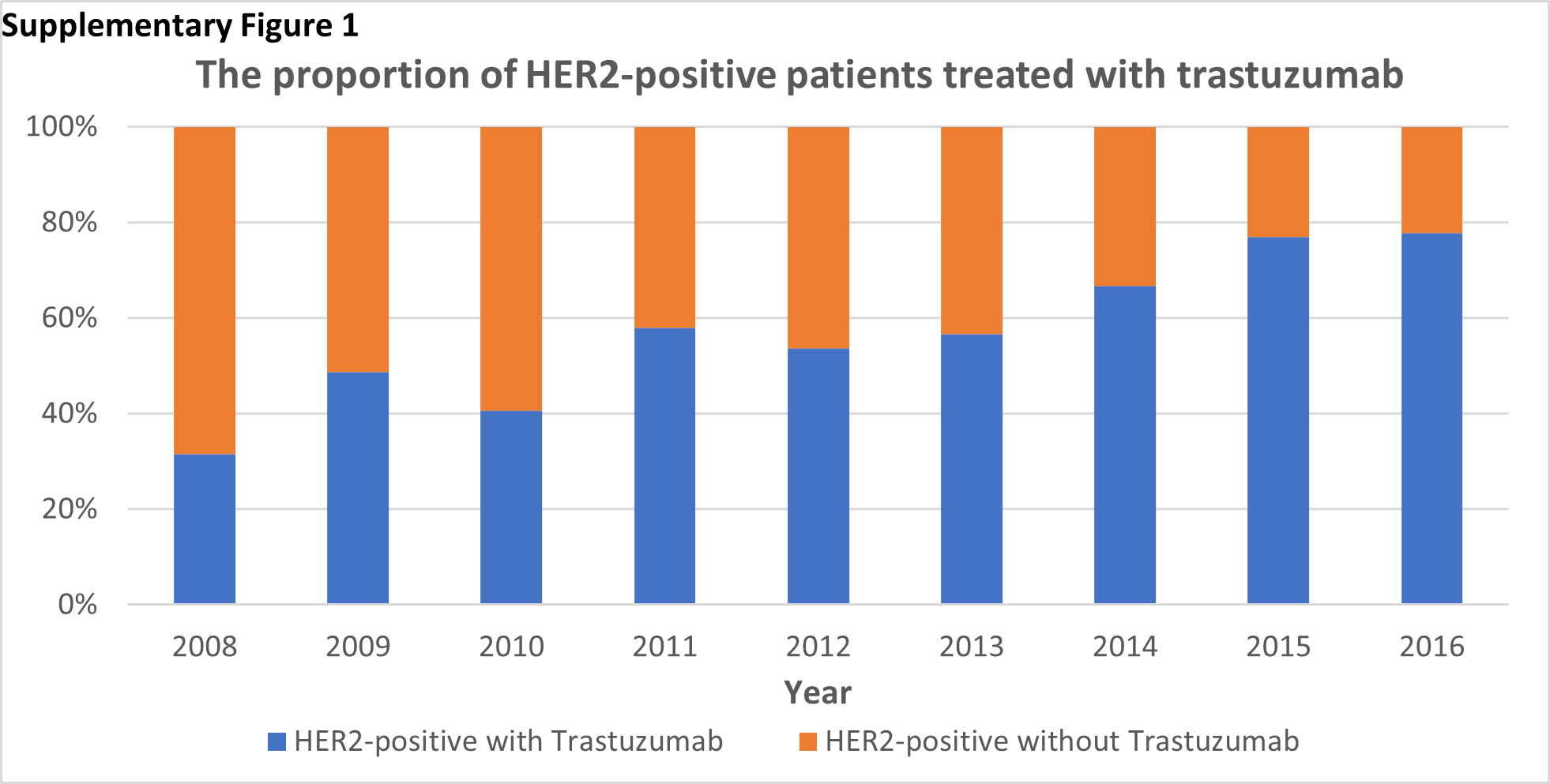

Supplement: Supplementary Figure 1 — The trends of the proportion of HER2-positive patients treated with trastuzumab over the study period. [file Image_1.tif]
